# The Most Common Type of HPV in Women with Atypical Squamous Cell of Undetermined Significance (ASCUS) in Pap Smear in Iran-Yazd

**Published:** 2015-12

**Authors:** Mojgan Karimi-Zarchi, Afsarosadat Tabatabaie, Alie Dehghani-Firoozabadi, Farima Shamsi, Maleknaz Baghianimoghaddam, Mandana Dargahi, Pouria Yazian, Shahnaz Mojahed

**Affiliations:** 1 Associate prof, Gynecological Oncology fellowship, Clinical research development, Shahid Sadoughi University of Medical Science, Yazd, Iran;; 2 Associate prof, Obstetrics & Gynecologist, Shahid Sadoughi University of Medical Science, Yazd, Iran;; 3 Obstetrics & Gynecologist, Shahid Sadoughi University of Medical Science, Yazd, Iran;; 4 Epidemiologist, Shahid Sadoughi University of Medical Science, Yazd, Iran;; 5 General physician, Shahid Sadoughi University of Medical Science, Yazd, Iran;; 6 Pathologist, Azad University of Medical Science, Yazd, Iran;; 7 Medical Student, Student Research Committee, Shahid Sadoughi University of Medical Sciences, Yazd, Iran;; 8 School of Nursing and Midwifery, Shahid Sadoughi University of Medical Sciences, Yazd, Iran

**Keywords:** HPV, high risk, low risk, cervical cancer, atypical squamous cells of undetermined significance, management, prevention

## Abstract

**Introduction:**

Cervical cancer is the third most gynecological cancer and one of the common causes of cancer death in women in Iran and the other developing countries. Human Papilloma Virus (HPV) is a known Risk factor in cervical cancer, but according to HPV deference types, the high risk and low risks differ.

**Material and method:**

We evaluate the most common high risk and low risk HPV type in 180 females with an atypical squamous cells of undetermined significance (ASCUS) results in pap smear in Gynecological Oncology Clinic in Shahid Sadoughi Hospital in Yazd, Iran within 2012 to 2014.HPV typing was done with polymerase chain reaction (PCR) method. The data obtained were recorded in a questionnaire and analyzed by SPSS software.

**Result:**

More common low risk HPV type in ASCUS patients was type 6 (63.6%) and then type 11 (36.4%). Type 16 was the most common high risk HPV type.

**Discussion:**

HPV DNA typing for better management of women With ASCUS is important and this study showed HPV type 16 is the most prevalent type in ASCUS patients. It seems the living region is important in HPV type distribution and Quadri-valant Vaccine can prevent cervical cancer in Iran because the most common low risk HPV is type6 and 11, and HPV 16 is the most common high risk HPV.

## INTRODUCTION

Cervical cancer is the fourth most common cancer in women and near 7.5% deathfrom cancer in females is due to cervical cancer worldwide (1-2).

Risk factors such as early age marriage, poor sexual hygiene, using hormonal contraceptives, sex working and… are important in cervical cancer incidence (3). In past decay, researchers work on “if the HPV infection sufficient factor in cervical cancer disease?” but it seems that this factor is necessary nor sufficient.

De Villiers EM explained more than 118 types of HPV that near the 40 types can infect the genital mucosa (4).

Different genotypes of the virus, based on ability to change the tissue as cancer, is divided into two categories: high and low risk. More common low risk types are HPV 11 and 6 that cause condyloma accuminata and benign genital warts. In high risk groups, HPV 16 and 18 are common as the etiology of cervical cancer (5).

This short paper is a part of a cohort study that explain the common HPV types in ASCUS patients in IRAN.

## MATERIAL AND METHOD

A clinical trial study was conducted among 180 female with an ASCUS results in pop smear. Patient selected randomly from women referred to Shahid Sadughi gynecologic clinic.

Patients with cervical cancer, were excluded from the study. Demographic data and patient’s medical history collected in a questionnaire. After getting informed consent, a sample was prepared for HPV reflex. HPV typing was done with PCR method, by single Laboratory and technique.

All patients, were called for repeating pop smear and biopsy for further research.

The collected data analyzed by SPSS21 software.

## RESULTS

180 patients enrolled in the study.

More common low risk HPV type in ASCUS patients was type 6 (63.6%) and then type 11 (36.4%).

Type 16 as the more common high risk HPV type was in 79.3% of patient and then respectively were Type 31 (8.6%), 45 (6.9%), 18 (3.4%) and 59 (1.7%) (Table 1).

**Table 1:** Distribution of HPV sample

HPV typing		Number

Low risk	6	7
		3.8
	11	4
		2.2
Total		11
		6.11
High risk	16	46
		25.55
	31	5
		2.7
	45	4
		2.2
	18	2
		1.1
	59	1
		0.55
Total		58
		32.22
No HPV		111
		61.66
Total		180
		100

After biopsy there was no association between biopsy result and HPV typing (*p*=0.095) (Table 2).

**Table 2:** Associateion of biopsy result and HPV typing

		HPV typing		Total
		High risk	Low risk	

Biopsy typing	CIN	16	5	21
	cancer	0	1	1
Total		16	6	22

## DISCUSSION

HPV infection is an Important risk factor for cervical cancer as is seen in 99% of CC patients (2, 6-8, 15, 16).

Our cases came from the group were referred to a gynecologist because of the previous ASCUS result in PaP smear.

Studies have shown, type 16 is common, more than 60% in CC. In other steps, type 18, 45 and 31 respectively, are 10-15%, 7% and 3% of HPV infection in CC patients (9-10, 17, 18).

Our study shows, type 16 is more common high risk type as others in ASCUS patients [then 31, 45 and 18]. In the more similar study done by *Clifford GM*, the prevalence of HPV 16 in ASCUS patients was 31% that in our study was 25.5%. Consistent with other studies, HPV type16 is the most common type in our specimens (10).

Wei H found 8.8% of LR-HPV in ASCUS patients (11) and Neilsen found 33.1% in such patients (12). Martin Pis showed, type 6 with 9.9% and type 11 with 3.9% in ASCUS patients (13). In our study, 6.11% LR-HPV was found in ASCUS patient that type 6 was 3.8% and type 11 was 2.2%.

As is seen, our results are near other studies. The present differences are contributed to differ epidemiological pattern in different region. Also the used primer system is important too. For example The GP5+/6+ primer set is less sensitive for HPV 53 detection, whereas MY09/MY11 is less sensitive for HPV 31 (13).

In Iran we don’t have many cases of cervical cancer but we have women with advanced cervical cancer because we don’t have mass screening program and vaccination isn’t as a routine primary prevention for all of girls within 9-26 years. Thus increasing knowledge of women for primary prevention and vaccination by qudrivalent (Gardazil) Vaccine can decrease cervical cancer (15-18). This paper help us for approval of this point that HPV type of 6,11 as low risk HPV and HPV 16 is the most common type of High risk HPV that is similar to the other part in the world.
